# Colorectal Carcinogenesis: A Cellular Response to Sustained Risk Environment

**DOI:** 10.3390/ijms140713525

**Published:** 2013-06-27

**Authors:** Kim Y. C. Fung, Cheng Cheng Ooi, Michelle H. Zucker, Trevor Lockett, Desmond B. Williams, Leah J. Cosgrove, David L. Topping

**Affiliations:** 1 CSIRO Preventative Health National Research Flagship, P.O. Box 10041, Adelaide, SA 5001, Australia; E-Mails: chengchengooi@gmail.com (C.C.O.); michelle.zucker@gmail.com (M.H.Z.); trevor.lockett@csiro.au (T.L.); leah.cosgrove@csiro.au (L.J.C.); david.topping@csiro.au (D.L.T.); 2 Sansom Institute for Health Research, School of Pharmacy and Medical Sciences, GPO Box 2471, Adelaide, SA 5001, Australia; E-Mail: des.williams@unisa.edu.au; 3 CSIRO Food Futures National Research Flagship, P.O. Box 10041, Adelaide, SA 5001, Australia

**Keywords:** ammonia, colorectal cancer, dietary protein, resistant starch, short chain fatty acid

## Abstract

The current models for colorectal cancer (CRC) are essentially linear in nature with a sequential progression from adenoma through to carcinoma. However, these views of CRC development do not explain the full body of published knowledge and tend to discount environmental influences. This paper proposes that CRC is a cellular response to prolonged exposure to cytotoxic agents (e.g., free ammonia) as key events within a sustained high-risk colonic luminal environment. This environment is low in substrate for the colonocytes (short chain fatty acids, SCFA) and consequently of higher pH with higher levels of free ammonia and decreased mucosal oxygen supply as a result of lower visceral blood flow. All of these lead to greater and prolonged exposure of the colonic epithelium to a cytotoxic agent with diminished aerobic energy availability. Normal colonocytes faced with this unfavourable environment can transform into CRC cells for survival through epigenetic reprogramming to express genes which increase mobility to allow migration and proliferation. Recent data with high protein diets confirm that genetic damage can be increased, consistent with greater CRC risk. However, this damage can be reversed by increasing SCFA supply by feeding fermentable fibre as resistant starch or arabinoxylan. High protein, low carbohydrate diets have been shown to alter the colonic environment with lower butyrate levels and apparently greater mucosal exposure to ammonia, consistent with our hypothesis. Evidence is drawn from *in vivo* and *in vitro* genomic and biochemical studies to frame experiments to test this proposition.

## 1. Introduction

Colorectal cancer (CRC) is a common internal malignancy in affluent countries and is appearing rapidly in developing countries with greater prosperity. It is the fourth most common cause of cancer-related deaths worldwide [1]. In Australia, it is the most frequent cause of cancer morbidity and mortality with over 14,000 diagnosed cases (13% of all cancers) and over 4000 fatalities in 2007 [2]. Similarly, more than 59,000 people die every year from CRC in the United States [1]. Epidemiological data show that there is a major geographical variation in the incidence of CRC, with populations in Africa and Asia showing lower risk [1]. This suggests that environmental factors are influential in carcinogenesis, a proposition supported by rapid temporal increases in CRC morbidity and mortality in countries such as Japan [3]. Nutrition has been identified as a potentially significant risk factor [4]. Specifically, diets that are low in fibre and unrefined grains, and high in energy (fat) and protein, are associated with increased risk of CRC [5]. This dietary pattern is established in developed countries, and becomes apparent in countries traditionally at low risk as they become more affluent. For example, in Singapore the age-standardised rates for CRC from 2003 to 2007 showed a 125% increase over the rates from 1968 to 1972 for males, and a 112% increase for females, with a notably sharp increase in the 40–45 year age group [6].

Population data also suggest that only a small fraction (possibly as low as 5%) of CRC cases are due to heritable factors [7]. Individuals who have two or more close relatives with CRC make up about 20% of all CRC patients, but only 5%–10% of cases actually develop from inherited genetic abnormalities. The greater number of cases (by far) is sporadic in origin where both genetic and environmental factors are important [8,9]. This implies that at least 80% of CRC are inducible and could be prevented with changes in diet and lifestyle. Therefore, it is important to understand the underlying mechanisms of the onset and development of sporadic CRC to formulate a rational dietary strategy to implement risk reduction.

There are abundant prospective cohort data linking dietary and lifestyle factors to CRC risk [10]. Exercise, whole grain dietary fibre consumption and aspirin [11–13] confer protection while cigarette use and greater consumption of red and processed meat increase risk [14,15]. Recent experimental data support the importance of environmental factors both in colorectal tumorigenesis and its possible prevention. Genetic damage is a pre-requisite for oncogenesis and it has been shown that diet alone increases colonic DNA damage in model animal species. Single and double strand DNA breaks were increased with dietary levels of protein such as casein, soy and red meat [16–18]. More recently, colonocyte telomere shortening was observed in rats fed a high protein diet, supporting a role for diet in early stages of carcinogenesis [19]. In these studies, the deleterious changes were reversed by the feeding of dietary fibre as resistant starch (RS) or arabinoxylan. Other rodent studies support the idea that dietary components (including fibre and n-3 polyunsaturated fatty acids) are protective [20,21], and that this protection may occur via moderation of inflammatory responses. However, the exact mechanism(s) of colorectal carcinogenesis remain ill-defined and the current models appear to be incomplete. In this paper, known models of colorectal carcinogenesis are reviewed in the context of documented risk factors, and how modification of these risk factors might act to promote or diminish oncogenesis.

We hypothesise a modified model of CRC development where oncogenesis is a random event reflecting a cellular response to a sustained risk environment. We suggest that this occurs as a result of continuous low grade exposure to carcinogens, paying particular attention to free ammonia. We suggest also that the focus on dietary fibre may have been misdirected. Resistant starch (RS), rather than non-starch polysaccharides (NSP), may be a key protector of CRC. This hypothesis acts as a potential route in CRC prevention at the individual and population level. We provide support of the hypothesis from the published literature.

## 2. Current Models of Colorectal Cancer

The current models for CRC are essentially linear in nature with a sequential progression from aberrant crypt foci and micro-adenomas, to adenomas and frank malignancy, via hyper-proliferation of the upper crypt cells [22,23]. The first step in the development of tumours from normal epithelium is usually taken to be the onset of dysplasia and single dysplastic crypts are thought to be the first histological manifestations of tumours. This has been described as the “adenoma to carcinoma hypothesis” and is often referred to as the conventional pathway to colorectal cancer. Vogelstein *et al.* provided a molecular basis for the adenoma to carcinoma sequence by describing the complex multi-step process in which cells accumulate genetic changes (especially gene deletions and activations) that control cell growth and differentiation [24]. With time, these accumulated errors coalesce, resulting in the neoplastic phenotype. The serrated pathway is also gaining acceptance as an alternate molecular pathway to CRC. In contrast to the lesions of the conventional pathway which harbour mutations in the APC gene, adenomas and tumours of the serrated pathway are characterised by mutation in the BRAF gene [25,26]. These factors have been synthesised in a model where the accumulation of changes (rather than their chronological order) determines histopathological and clinical characteristics of the colorectal tumour [27].

These views of CRC development may be true but do not explain the full body of published knowledge and tend to discount some influences. Specifically, CRC is virtually unknown in some societies eating “traditional” plant based diets, but appears quite rapidly when such populations become more affluent (and change their dietary habits). Indeed, much of the interest in the potential of diet to prevent chronic disease can be traced to early observational studies with native populations in whom those diseases were so rare as to be remarkable [28]. These low risk diets are generally low in total and saturated fat, high in complex carbohydrates (starch and NSP, major components of dietary fibre), and low in animal products.

Specific dietary components have been linked to altered risk. For example, population studies have linked consumption of red and/or processed meat to greater risk [29,30]. Higher intakes of fat are also associated with greater risk [31,32]. Dietary fibre is one of the factors where the expectations of a strong protective effect were greatest, based to some degree on the observational studies by Burkitt *et al.* who compared diet and risk in native black African populations [33]. However, the reality has proved to be more complex than anticipated. Fibre is an established faecal bulking and laxative agent in proportion to intake, and an inverse relationship has been demonstrated between stool mass and cancer risk [10]. Part of this protection is thought to be due to dilution of carcinogens leading to less exposure to the colonocytes [34]. Faecal outputs by populations at low risk are generally high. However, while some prospective studies have identified an important protective role for fibre [10,35,36], other studies have failed to show any substantial effect [37]. There is also the paradox of high and rising CRC rates in Australia, despite population-wide intakes of (largely cereal) fibre [38]. These discrepancies can be resolved if one considers the food components which actually contribute to total dietary fibre intakes and which could protect against CRC through altering the colonic environment.

## 3. Diet and the Normal Colonic Environment

The adult human large bowel is home to a large and complex bacterial eco-system comprising of 13 genera, and each individual has several hundred species of these genera, with a particular combination of predominant species that is distinct from that of other individuals [39]. Advances in molecular technologies have assisted greatly in understanding the complex structure and dynamics of the bacterial population [40–42]. It is well-established that anaerobic organisms predominate and that they metabolise undigested nutrients escaping from the small intestine, plus endogenous small and large intestinal secretions. Thus, the colonic environment reflects the interaction between these nutrients with the microbiota and their metabolic end products.

Digestion of the major nutrients in the human small intestine is incomplete, especially that of complex carbohydrates [43]. Humans possess only one intrinsic polysaccharidase, α-amylase, which can hydrolyse only one polysaccharide (starch). Dietary fibre consists principally of NSP which resists small intestinal enzymatic hydrolysis completely such that they pass into the large bowel quantitatively. There is also strong evidence that the ileal digestibility of starch is less than 100% and a fraction, depending on the nature of the food and an individual’s characteristics, pass into the large bowel [44]. This fraction is termed RS. The importance of NSP to colonic function is recognised. However, it is becoming apparent that RS may be as (or even more) important.

Examination of a traditional African (low risk) diet shows that their dietary fibre consumption is actually lower than that of some high risk westernised diets. However, their diet contains more starch, largely as whole grain maize [45]. Whole grain starchy foods are generally higher in RS than refined ones through the physical barrier presented by the bran. However, cooking practices of the Africans seem to be more important as it favours the generation of RS through retrogradation [46]. Foods are cooked by heating in water, which leads to gelatinisation and greater digestibility in the small intestine. However, it is the African practice to store cooked porridge for some time, allowing the starch chains to reassociate (retrograde). This leads to the formation of starch that is not digested in the small intestine and that enters the large bowel, *i.e.*, RS.

### 3.1. Fermentation of NSP and RS

Both NSP and RS are subject to fermentation by the human large bowel microbiota which obtains energy for maintenance and growth. However, while a variable fraction of NSP is fermented, that of RS seems to be largely complete in most individuals. In adults, short chain fatty acids (SCFA), principally acetate, propionate and butyrate, are the major metabolic end-products of this fermentation [47]. They are the main anions in normal large bowel digesta and are critically important for colonic function through a range of actions including lowering of pH (which induces apoptosis of cancerous cells and protects against overgrowth by pathogenic micro-organisms), stimulation of fluid and electrolyte absorption, and enhancement of colonic blood flow through relaxation of resistance vessels in the vasculature. SCFA are absorbed with less than 10% of total production appearing in faeces [48]. Of the major SCFA, acetate appears to have no specific properties above being a metabolic intermediate. In contrast, propionate and (more particularly) butyrate are thought to play a pivotal role in promoting normal colonic function and preventing serious disease [49].

A nutritional study with staled maize porridge (as consumed by native Africans) showed that it favoured large bowel bacterial butyrate production compared with fresh porridge [45]. This, plus a higher basal total SCFA and butyrate excretion, provides an explanation for the improved large bowel health in this population despite a lower fibre intake [46]. The Africans may have consumed a lot of NSP, but as intakes of the fibre components decreased in modern Africans diets, those of RS appear to have been maintained. While native African cooking practice is unchanged, this does not appear to be so for African Americans who are at a very high risk of CRC and also consume relatively little NSP and RS [50].

Interest in the particular attributes of butyrate is based on an extensive body of literature from *in vitro* and *in vivo* animal and human studies. Animal studies have shown that butyrate infusion relaxes resistance blood vessels in the large bowel mesentery [51]. This would have the effect of increasing tissue perfusion with blood and, hence, oxygenation. Butyrate also has a concentration-dependent, biphasic action on the large bowel musculature. At low concentrations (as low as 3 mM), butyrate infusion into the large bowel lumen relaxes the muscles. At higher concentrations, contraction is stimulated [52]. Propionate has similar effects, albeit at much high concentrations. It has been shown that butyrate is a preferred metabolic substrate for colonocytes, especially those isolated from the distal region. Butyrate is oxidised in preference to other substrates and suppresses the utilisation of glucose, glutamine and other fuels in isolated colonocytes [53]. Increased oxidative activity, and hence greater cation absorption, is thought to account for the greater uptake of Na^+^ and K^+^ which is thought to account for the greater water salvage observed when large bowel SCFA are increased through the feeding of RS. This effect has been demonstrated quite clearly in humans with cholera toxin-induced diarrhoea, with a substantial shortening of time to recovery and diminution of fluid loss. It was thought formerly that cation recovery was limited to Na^+^ and K^+^ but there is increasing evidence that colonic salvage of Ca^2+^ and Mg^2+^ is also increased [51,54].

Butyrate has a pK_a_ of 4.82 and therefore is present predominantly in the ionised form in the human colonic environment. For instance, at pH 7 there is only 0.16% unprotonated butyrate compared to 2% at pH 6.5. Nonetheless, due to its small molecular size, it can enter colonocytes via both active transport and passive diffusion (to a lesser extent at higher pH) pathways. In situations with high SCFA, luminally-derived butyrate is the preferred metabolic fuel for colonocytes via β-oxidation to produce energy for proliferation of the normal colonic mucosa [48,55,56]. Fibre serves as a bulking agent and shortens transit time, hence reducing the exposure of the colonic epithelium to carcinogens. However, there is also an anatomical dimension to large bowel physiology and disease risk. Fermentation predominates in the caecum and proximal colon due to the greater availability of substrate and this is the region where SCFA levels are highest and disease risk is lowest. With passage of the faecal stream, SCFA levels fall (due to uptake of SCFA and depletion of substrate) and pH values rise. The distal colon is the site of greatest risk of CRC and Cats *et al.* [55] have drawn attention to this fact, suggesting that lack of SCFA predisposes the distal colon to this malignancy.

### 3.2. Protein Fermentation and Ammonia Production

Bacterial degradation of colonic nitrogenous substrates, such as deamination of dietary protein residues, intestinal secretions from shed epithelial cells, and bacterial hydrolysis of urea in the hindgut produces ammonia [57–59]. Other potentially toxic compounds such as phenols, cresols and hydrogen sulfide are produced from protein fermentation. Experimental studies have linked the cytotoxicity of faecal water to cancer risk through these and other metabolites [60]. However, it is free ammonia that is the focus of this paper. It is the form of nitrogen in the body that is most toxic and most readily absorbed by cells, and the role of ammonia in gastric mucosal damage induced by *Helicobacter pylori* (*H. pylori)* is well recognised [61–63]. Total ammonia (*i.e.*, NH_3_ + NH_4_^+^) concentrations in human faeces and in the digesta of model animal species consuming western-type diets are in the order of 3–10 mM [64,65]. Free ammonia diffuses readily and can be absorbed from the large bowel lumen into colonocytes, but it is well established that NH_4_^+^ is not absorbed. The pK_a_ value of ammonia is 9.24, so that in a normal or low risk colonic environment (pH < 7), a very large proportion is present as NH_4_^+^. This would leave only a small fraction as free ammonia to be absorbed by non-ionic diffusion, a process that is greatly enhanced by a gradient from higher to lower pH [55,60,66].

**Figure 1:**
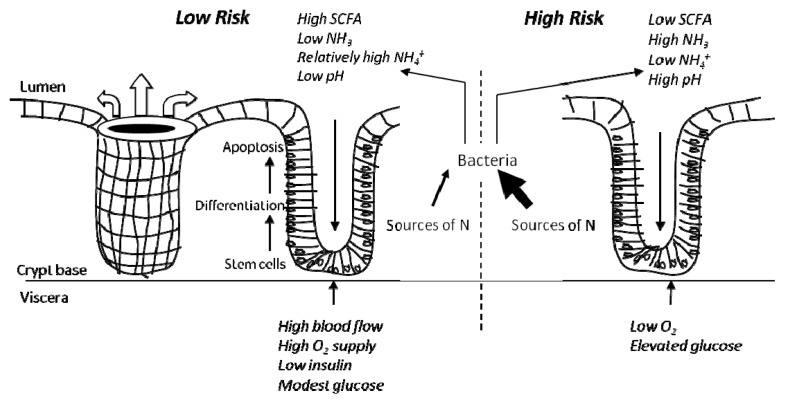
Low risk versus high risk colonic environment. A low risk colonic environment is characterised by well perfused mucosa and relatively high luminal concentration of SCFA, low levels of ammonia, and has low pH. In this low risk environment, mucosal cells utilise butyrate as the primary energy source and have low requirement for glucose. Conversely, a high risk environment is higher in pH and ammonia and low in SCFA. Mucosal cells adapt to these conditions, and acquire epigenetic and genetic changes to survive, predisposing to tumorigenesis.

Diets that are high in fermentable fibre, in particular RS, and low in fat and protein lead to an environment in the colon which is considered low risk for the development of CRC [10]. Experimental studies in humans and animals have shown that this gives a colonic environment which is relatively high in SCFA and of low pH, leading to a low level of free ammonia and other basic cytotoxins. The mucosa itself is well perfused, giving high oxygenation, while the availability of SCFA spares glucose utilisation. There is strong evidence that O_2_ supply is critical for hepatic metabolism, especially glucose homoeostasis, and there is evidence also that the entero-pancreatic axis may be involved in CRC risk with high insulin and insulin-like growth factors being implicated [67]. Animal and human studies suggest that fermentable carbohydrates improve blood glucose control so that it is possible that insulin may also be low in this scenario. Populations with a low risk of colonic cancer have been shown to have lower faecal pH than in higher risk groups [66]. Figure 1 illustrates this situation and our proposed risk environment.

## 4. Colonic Environment for Risk of Colorectal Cancer and Related Problems

### 4.1. The High Risk Environment

One characteristic of the gastrointestinal mucosa is that it undergoes rapid replication and turnover, requiring a readily available supply of nutrients for tissue synthesis. Hence, these rapidly regenerating tissues should be very responsive to dietary alteration, especially under marginal substrate availability [68].

**Figure 2:**
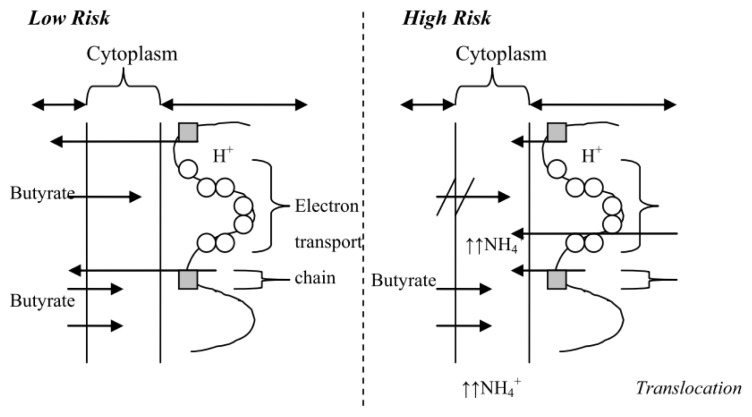
Cellular environment of low risk *versus* high risk of CRC.

We hypothesise a CRC model supporting the view whereby diets which are low in fibre and high in digestible energy and also proteins lead to a colonic environment considered at high risk of developing CRC. This environment (Figure 2) is low in SCFA and subsequently higher in pH, has higher concentrations of ammonia and decreased oxygen supply as a result of lower colonic mucosal blood flow, all leading to a greater exposure of the colonic epithelium to carcinogens. We propose that CRC is actually a response by the cell population seeking to manage local environmental conditions, and constant exposure to low but significant levels of free ammonia is a key event. Such adaptation occurs in the stomach for *H. pylori* where it synthesises a urease enzyme to create an alkaline environment protecting the organism from the bactericidal effect of acid [61].

Models of tumorigenesis suggest that mutations acquired by tumour cells are not a direct impact of external DNA damaging agents but are generated by the cell itself as a result of a mutation response [22,69]. This response has the characteristics of initiation of error-prone cell cycle progression and an increased rate of mutation [70]. As the cells adapt and evolve to survive in altered environments induced by dietary factors, via the “Darwinian” survival of the fittest, these cells become susceptible to genetic mutations and evolve into cells not subject to normal controls. We have adopted this idea and propose that as colonocytes progress from normal through to malignant, in response to the environment, they adapt to the new environment so efficiently that they eventually become unable to survive in the previous or “normal” environment. Thus, when CRC cells are exposed to high concentrations of butyrate and other SCFA, they are not able to survive and thus undergo apoptosis or programmed cell death [71–74]. Epigenetically reprogrammed and transformed CRC cells faced with altered environments such as shortage of oxygen and nutrients, in this case butyrate as the energy source, can easily express genes to increase mobility to allow migration and proliferation into new environments.

### 4.2. Influence of Diet and *in vivo* Studies

There are experimental data *in vivo* to support the model which we have proposed, some of long standing. When 2.5–5 g three times daily of dietary supplementation with fermentable fibre are taken for 30 days, cirrhotic patients were associated with significantly reduced circulating levels of ammonia and faecal pH, and significantly increased circulating levels of SCFA [75]. Ammonia favours the growth of cancerous cells over healthy cells *in vitro* [57]. Lin *et al.* [56] showed that the life span of colonocytes, due to mucosal cell damage and altered DNA synthesis, are shortened by ammonia concentrations (35 mM was used in the studies) found under normal dietary conditions. Ammonia is thought to be involved in the colonic carcinogenic response, where the same group showed that the highest ammonia concentrations and luminal pH were found in the region of colon where cell proliferation and the incidence of polyps and cancer are highest [56,76]. High ammonia has been shown to increase inflammatory lesions in rats, which are established precursors in animal models and humans to the development of CRC. It has also been shown to increase cell proliferation making neoplastic transformation more efficient. The enhanced proliferation may increase errors in DNA copying and unmask latent DNA changes caused by earlier mutations [57].

Toden *et al.* [18] reported that rats fed with higher levels of dietary animal protein (as casein or red meat) and dietary plant protein (as soy) manifested increased colonocyte DNA damage. Feeding the rats with RS attenuated the damage, and increased the large bowel SCFA pool hence lowering faecal pH, which reflected caecum pH [18]. Further data from rats and pigs confirmed that fermentable fibre opposed colonocyte genetic damage induced by Western-type diets [16,17]. Le Leu *et al*. also demonstrated that rats fed with RS were protected against intestinal tumorigenesis and RS decreased the tumour-promoting effects of indigestible protein (in particular digestion-resistant potato protein) on intestinal tumorigenesis in these rats [17]. Others have shown that both dietary whey and soy proteins altered the global gene expression profiles of colonocytes in rats with azoxymethane-induced colon tumours [77]. In rats fed with high amylose maize starch (HAMS) it was found that the dietary resistant protein source had a substantial influence on the fermentation products of HAMS. They suggested that these resistant proteins (rice, potato, soy and casein proteins) may alter the relative proportion of the caecal microbiota through the supply of nitrogen to the caecum and thus causing the differences in fermentation profiles of high amylose starch [78].

Duncan *et al.* [79] reported a large, statistically significant drop in faecal ammonia and SCFA (including butyrate) when healthy, obese volunteers consumed a high protein, low carbohydrate diet (30% protein, 4% carbohydrate, 66% fat as calories) as compared to an energy maintenance diet (13% protein, 52% carbohydrate, 35% fat). The lowering of SCFA is predictable but it would have expected that faecal ammonia would rise through diminished microbial utilisation. This study supports strongly our proposed model where the high risk colonic environment would facilitate free ammonia mobilisation into the cell and hence a decrease in excreted ammonia. The high risk colonic environment in this study would be high ammonia, low SCFA and high pH due to high protein, low carbohydrate and high fat diet. This, as explained previously, in a situation with high luminal ammonia, ammonia will be able to cross the mucosa barrier and only be absorbed by the colonocytes at alkaline pH. The molecules then diffuse into the cytoplasm following the high to low concentration gradient. This leads to a more alkaline environment in the cytoplasm as more and more ammonia is present. In order to neutralise the environment, mitochondria will pump out proton molecules and ionise ammonia molecules into ammonium ions.

Lewin *et al.* [80] showed that there was a consistent and statistically significant increase in faecal *N*-nitrosocompounds (NOC) with a red meat diet in 21 volunteers. This study has shown that faecal NOC from red meat is able to form alkylating DNA adducts (specifically O^6^carboxymethylguanine adducts). If these and other related adducts are formed but not repaired, and cause mutation in key oncogenes and/or tumour suppressor genes, these events may explain the association of red meat with CRC [80,81].

### 4.3. *In vitro* Data to Support a “High Risk” Environment

A number of alterations to normal protein expression have been demonstrated in CRC cells both *in vitro* and *in vivo* when adapted to the “high risk” environment [82,83]. Additionally, CRC cells have been shown to have a down-regulation of the primary butyrate transporter, monocarboxylate transporter-1 (MCT-1). In conjunction with this, up-regulation of the high affinity glucose transporter, GLUT-1, and down-regulation of GLUT-2 (low affinity) would enable the cells to take up and utilise glucose efficiently and ensure their growth and survival in the absence of their conventional energy source, butyrate [84]. These data correlate with the hypothesis that there is a switch from butyrate to glucose as a preferential fuel source [53,84]. Recently, Donohoe *et al.* linked the effects of butyrate metabolism and the Warburg effect to epigenetic changes in colonocytes and hypothesized that this change in metabolic profile drives tumorigenesis in a “high risk” environment [85]. This has implications for colonic stem cells that undergo proliferation and differentiation. In a “high risk” environment, these cells have the potential to accumulate genetic and epigenetic changes that may contribute to tumorigenesis, especially if these result in somatic mutations of oncogenes or silencing of key tumour suppressor genes. *In vitro* studies have also shown that in the presence of butyrate, colon carcinoma cells (e.g., HT-29 cells) acquire a differentiated phenotype through replacement of glucose for butyrate as the main carbon source with alterations in the transporter expression [84,86]. This clearly shows that a colon cancer cell has the ability to reverse its cellular transport system under the appropriate conditions. This opens the prospect of early diagnosis and prevention of CRC as epigenetic alterations may be reversible and correction of the cellular environment at this stage with proper treatment or by changing lifestyle factors [65,69].

De Silanes *et al.* [87] established a butyrate-resistant human adenocarcinoma cell line (BCS-TC2.BR2, growing continuously in 2 mM butyrate) from non-tumorigenic BCS-TC2 cells. The BCS-TC2.BR2 cells were resistant to stress-induced apoptosis and revealed a phenotype where their survival rates after glucose deprivation and heat shock were higher than those of the parental cells. The same group also reported that attaining such a resistant phenotype was accompanied with the acquisition of tumorigenic capacity where they inoculated BCS-TC2.BR2 cells into nude mice and these mice developed tumours [87]. This clearly demonstrates the ability of cells to adapt to the new environment for survival and this has been further supported by identification of mechanisms that potentially mediate the development of butyrate-resistance [88–91]. The data suggest that environmental pH, and subsequently, the overall environmental condition influences the growth and survival of cells. Cells need to undergo adaptation to the new environment to ensure survival in the sustained stress environment. As the colonocytes adapted to the “high risk” environment, the cells become more specific and therefore sensitive to changes in the intestinal environment. Butyrate resistance may also help to explain the recent findings from the CAPP2 study where a supplement of RS failed to alter polyp recurrence in patients with the Lynch syndrome [92]. It must be noted that the supplement was quite small and no faecal SCFA data were reported, but it is possible that butyrate may be ineffective in cancers where genetic predisposition is the dominant factor.

## 5. Conclusions

The conventional models of CRC suggest a linear adenoma to carcinoma sequence. However, the emerging evidence suggests an alternative scenario which is a response to sustained exposure to a hostile environment. This environment is the result of dietary intakes which do not favour healthy homeostasis of normal colonocytes. This hypothesis clearly explains the “butyrate paradox” phenomenon which has been observed for many years. In normal healthy large intestine, butyrate is a preferred energy source. However, in the shortage of butyrate, attributed partly by “Western diet”, glucose is substituted as the energy source for survival of these colonocytes. As they evolve to adapt to the new conditions, genetic manipulations are initiated with subsequent loss of function of critical genes and eventual loss of ability to undergo programmed cell death. These cells may therefore be considered as “normal” so that if the initial or healthy environment has been re-introduced, for example, by the presence of higher concentrations of butyrate, they will not be able to adapt rapidly due to their altered genetic make-up. Hence, they will undergo butyrate-induced apoptosis, as seen in many *in vitro* and animal studies.

Undigested dietary carbohydrates, especially RS, induce a low risk colonic environment by acting as a source of SCFA (which lowers luminal pH, increases abundance of butyrate-producing bacteria, serves as an energy source, increases blood flow and relaxes muscle in the large bowel) and bulking agents (which shorten intestinal transit time and hence reduce luminal exposure to carcinogens, in particular free ammonia). Diet alone (as dietary protein) has been shown to induce genetic damage in the large bowel [18], most probably due to sustained exposure to free ammonia. Bajka *et al.* found that ammonia levels in the caecal digesta were increased by RS in rats fed with high protein diet and the levels correlated negatively with digesta pH [93]. They suggested diminished exposure of colonocytes to this cytotoxic agent. Recent data provide evidence that RS reverses protein-induced colonocyte DNA damage in animal models, by altering the colonic environment [17,18,77,93]. Although the dietary fibre intake in native African populations has fallen due to urbanisation, their RS intake remains unchanged. Paradoxically, the incidence of CRC in these populations remains low despite reduced dietary fibre in their daily diets [94]. Human subjects on high protein, low carbohydrate diets are the best candidate to test our proposed CRC model. This model provides a means to prevent CRC in populations at risk.

Our proposed model may help to explain the inconsistencies with the role of dietary fibre in CRC prevention. It must be emphasised that this model is aimed at providing a basis for the prevention of sporadic CRC. The recent study in patients with the Lynch syndrome [92] underscores the possibility that our hypotheses does not apply to genetic predisposition to CRC. One way to test this proposal is to obtain the RS contents in various published human trials, and those data can be re-analysed to establish the correlation between RS and CRC risk.
